# Chemical Composition, In Vitro Antioxidant Potential, and Antimicrobial Activities of Essential Oils and Hydrosols from Native American Muscadine Grapes

**DOI:** 10.3390/molecules24183355

**Published:** 2019-09-15

**Authors:** Vasil Georgiev, Anthony Ananga, Ivayla Dincheva, Ilian Badjakov, Velizar Gochev, Violeta Tsolova

**Affiliations:** 1 Laboratory of Applied Biotechnology, The Stephan Angeloff Institute of Microbiology, Bulgarian Academy of Sciences, 139 Ruski Blvd., 4000 Plovdiv, Bulgaria; 2 Center for Viticulture and Small Fruit Research, College of Agriculture and Food Science, Florida A&M University, Tallahassee, FL 32317, USA; anthony.ananga@gmail.com (A.A.); violeta.tsolova@famu.edu (V.T.); 3 Food Science Program, College of Agriculture and Food Sciences, Florida A&M University, Tallahassee, FL 32307, USA; 4 AgroBioInstitute Agricultural Academy, 8 Dr. Tsankov Blvd., 1164 Sofia, Bulgaria; ivadincheva@yahoo.com (I.D.); ibadjakov@gmail.com (I.B.); 5 Department of Biochemistry and Microbiology, Faculty of Biology, Plovdiv University “P. Hilendarski”, 4000 Plovdiv, Bulgaria; vgochev2000@yahoo.com

**Keywords:** *Vitis rotundifolia* Michx., volatile compounds, grape skins, antifungal activity

## Abstract

Essential oils and hydrosols of two cultivars of muscadine grapes (*Muscadinia rotundifolia* (Michx.) Small.) were obtained by hydro-distillation of flowers and berry skins. Twenty-three volatile compounds were identified in essential oils from the muscadine flowers, and twenty volatiles in their corresponding hydrosols. The composition of volatiles in berry skins differed significantly from that of the vine flowers. The antioxidant potential of investigated essential oils and hydrosols was evaluated using five in vitro assays: DPPH (2,2-diphenyl-1-picrylhydrazyl) method, TEAC (Trolox equivalent antioxidant capacity), FRAP (Ferric reducing antioxidant power), CUPRAC (cupric ion reducing antioxidant capacity), and NO (nitric oxide radical scavenging assay). The essential oils from the flowers of both cultivars showed the strongest antioxidant power, whereas the hydrosols were the significantly less active. All investigated essential oils showed very weak antibacterial activities against *Bacillus cereus*, *Staphylococcus aureus*, *Escherichia coli*, and *Pseudomonas aeruginosa*. However, the essential oils from the flowers of both cultivars showed moderate antifungal activities against *Candida albicans*, which were stronger for the oil from “Carlos” (white muscadine cultivar). To the best of our knowledge, this is the first report on obtaining and characterizing essential oils and hydrosols from muscadine grapes. This study demonstrated the variations in aromatic compounds accumulated in flowers and mature berry skins of muscadine grapes, and evaluated their possible antioxidant and antimicrobial activities. The presented results will be the base for future research, focused on a better understanding of the molecular and regulatory mechanisms involved in aromatic compound biosynthesis and accumulation in muscadine grapes.

## 1. Introduction

Essential oils are mixtures of volatile plant compounds with distinctive scents. In addition to their traditional applications as fragrance ingredients in cosmetic and perfumery products, in the past decade, a growing number of essential oils have been studied for possible applications in medicine, aromatherapy, for extending the shelf life of food products, or in agriculture as bio-pesticides [1,2,3,4,5]. Usually, at room temperature, most essential oils are liquids which are insoluble or have very low solubility in water [6]. Essential oils can be obtained from aromatic plants by different methods; steam distillation, hydro-distillation, solvent extraction, Soxhlet extraction, simultaneous distillation–extraction, solvent-free microwave extraction, and halocarbons (Freon) extraction have all been reported [7,8]. However, hydro-distillation is the most popular classical method. By this method, both essential oil and hydrosol can be obtained. The hydrosol is the distilled water phase after separation of essential oil [9]. Even though hydrosols are a by-product of the essential oil distillation process, they still contain certain amounts of polar or partially miscible with water volatiles, and can possess valuable aromatic properties or biological activities [10]. Most of the essential oils showed remarkable antioxidant and/or antimicrobial activities, which made them attractive objects for the development of natural additives for improving food quality, or for inclusion as active substances in cosmeceuticals [6,11,12,13].

Grape is one of the most important fruit crops worldwide. Because of the formation of wine aroma, study of the volatiles of grapes is very important in viniculture. To date, most research efforts have been focused on investigating the volatile compositions of grape berries, juices, wines, and musts, and very little is known about volatiles in the flowers or other parts of the vine [8,14,15,16,17]. However, most investigation of volatile compounds in grapes has involved the application of headspace analysis, which does not allow the collection of essential oils and cannot be used to evaluate biological activities.

Muscadine (*Muscadinia rotundifolia* (Michx.) Small.) was the first native grape species cultivated in the United States [18]. Muscadine grapes differ significantly from European grapes (*Vitis vinifera* L.) not only by their physiology, phytochemical composition, disease tolerance, flavor, and taste, but also by the number of their somatic chromosomes (40 (*n* = 20) in muscadine vs. 38 (*n* = 19) in European grapes) [19,20]. Muscadines are well known for their health promoting effects because of their unique mix of phytochemicals with remarkable biological activities [18]. However, very little is known about the volatile compounds, responsible for formation of the specific muscadine aroma [21,22,23]. To the best of our knowledge, there has been no research to date focused on characterization of the chemical composition and biological activities of essential oils and hydrosols from the muscadine grape.

In this study, essential oils and hydrosols from flowers and berry skins of two muscadine cultivars, the red *Muscadinia rotundifolia* (Michx.) Small., cultivar “Noble”, and the yellow *Muscadinia rotundifolia* (Michx.) Small., cultivar “Carlos”, were obtained by hydro-distillation. The chemical compositions of obtained essential oils and hydrosols were determined using the gas chromatography-mass spectrometry (GC-MS) technique, and their antioxidant potential and antimicrobial activities were evaluated.

## 2. Results and Discussion

### 2.1. Essential Oil and Hydrosol Compositions

**Figure 1:**
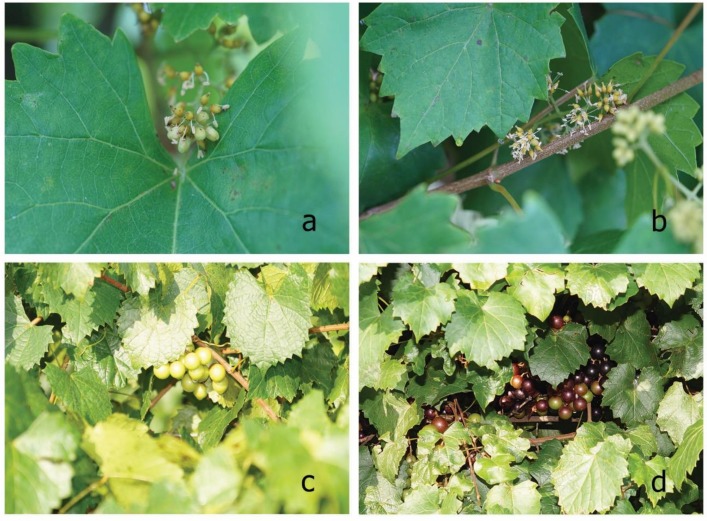
Flowers (**a**) and ripe berries (**c**) of yellow *M. rotundifolia* (Michx.) Small. cultivar “Carlos”, and flowers (**b**) and ripe berries (**d**) of red *M. rotundifolia* (Michx.) Small. cultivar “Noble”.

Flowers and ripe berries from red cultivar, Noble, and yellow cultivar, Carlos, of muscadine grape were collected and used for the distillation of essential oils (Figure 1).

Flowers from both cultivars were collected during their blooming phase. The maturity of the muscadine berries was evaluated on the basis of their pH, titratable acidity, and sugar contents. The berries from the Carlos cultivar were collected when they reached pH 2.99, a titratable acidity (TA) of 4.3 g/L, and a sugar content of 13.49° Brix. Similarly, berries from the Noble cultivar were collected when they reached pH 3.05, a titratable acidity of (TA) of 4.2 g/L, and a sugar content of 14.30° Brix. The berry skins were separated from the pulps and used for distillation.

The obtained yields for essential oils from Carlos flowers and Noble flowers were 0.56 ± 0.002% (*w*/*w*) and 0.90 ± 0.048% (*w*/*w*) (the dry content of fresh flowers before distillation was 33.98% for Carlos and 31.78% for Noble). The obtained yields for essential oils from the Carlos and Noble berry skins were 0.012 ± 0.0001% (*w*/*w*) and 0.010 ± 0.0007% (*w*/*w*) (the dry content of berry skins before distillation was 18.91% for Carlos and 19.66% for Noble). The volumes of collected hydrosols for all oils were equal—100 mL. The lower oil yields obtained from muscadine grape berry skins define them as an ineffective source for commercial hydro-distillation of grapevine volatiles.

The qualitative composition of the oils and hydrosols is presented in Table 1. In total, 23 volatile compounds were identified in essential oils from muscadine flowers (accounting for 81.66–83.79% of TIC), and 20 volatiles (accounting for 93.46–93.46% of TIC) were found in their corresponding hydrosols (Table 1). The essential oils from berry skins showed a significantly different chemical profiles, where 20 volatiles were identified in the oils (accounting for 67.72–80.81% of TIC), and 7 compounds (accounting for 94.77–95.23% of TIC) were found in their corresponding hydrosols. The major compounds (higher than 4%), found in essential oils from flowers of both cultivars were similar, with only slight variations in the amounts: valencene (39.71% in Noble and 34.32% in Carlos), germacrene D (4.48% in Noble and 6.94% in Carlos), α-selinene (3.28% in Noble and 4.29% in Carlos), and α-cadinol (2.86% in Noble and 4.30% in Carlos). On the other hand, the chemical compositions of flower hydrosols differed significantly from those of the flower oils. The major identified compounds were: α-terpineol (12.22% in Noble and 10.39% in Carlos), β-linalool (10.73% in Noble and 12.44% in Carlos), 4-hydroxy-3-methylacetophenone (18.94% in Noble and 6.56% in Carlos), 3,4,5-trimethoxytoluene (7.42% in Noble and 4.65% in Carlos) and 1,3,5-trimethyoxybenzene (5.04% in Noble and 3.09% in Carlos). Moreover, compounds such as 4-hydroxy-3-methylacetophenone, 3,4,5-trimethoxytoluene, 1,3,5-trimethyoxybenzene, elemicin, nerolidol, ledol, and juniper camphor were detected only in hydrosols but not in the essential oils from muscadine flowers. Due to the significant differences in the compositions of the essential oil and their respective hydrosols, different biological activities were expected.

The volatile compositions of essential oils from muscadine berry skins differed significantly from those of the essential oils from muscadine flowers (Table 1). The major identified compounds in essential oils from berry skins were: α-terpineol (59.43% in Noble and 45.42% in Carlos), β-linalool (5.95% in Noble and 8.69% in Carlos), *allo*-ocimene (0.23% in Noble and 14.05% in Carlos), and myrcenol (0.08% in Noble and 5.10% in Carlos). The last two compounds presented in significantly higher amounts in Carlos berry skins. The absence of valencene and germacrene D (the major volatiles in flowers oils) could be explained by the transcriptional regulation of (+)-valencene synthase and (−)-germacrene D synthase gens, of which the expression levels have a maximum in grapevine flower buds, but have been found to decrease during the early stages of fruit development [24].

Similarly to the hydrosols from muscadine flowers, the major compounds in berry skins hydrosols were α-terpineol (72.83% in Noble and 65.41% in Carlos) and β-linalool (6.50% in Noble and 7.62% in Carlos) (Table 1). A recent headspace-SPME-GC-MS study on grape berries of *Vitis vinifera* L. cultivar “Baga” showed that the amounts of α-terpineol and linalool in berries were increased during ripening and decreased in post-maturation stages [25]. In our research, we demonstrated that in muscadine grapes, α-terpineol and linalool remained the major volatile compounds in the mature berries. We also found a relatively high amount of *allo*-ocimene in berry skins of the Carlos cultivar. It has been reported that *allo*-ocimene enhances resistance against the necrotrophic fungus *Botrytis cinerea* in *Arabidopsis thaliana* [26]. The observed high concentrations of *allo*-ocimene in Carlos grape berry skins could be one of the phytochemical factors responsible for the increased disease resistance of this muscadine cultivar. However, more research is needed to confirm the role of this volatile compound in muscadine defense.

**Figure 2:**
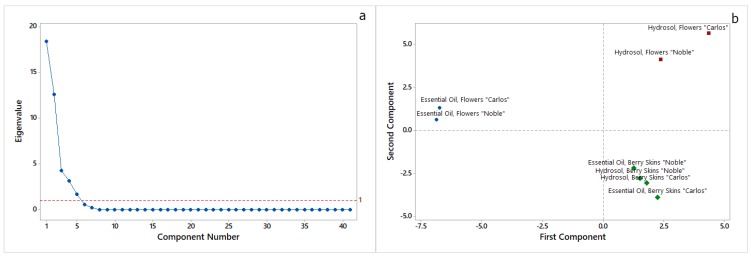
Scree plot (**a**) and score plot of the first two principal components (**b**), identified by principal components analysis (PCA) of volatile compounds in essential oils and hydrosols from two *M. rotundifolia* (Michx.) Small. cultivars.

Principal components analysis (PCA) was used to study the main sources of variability between the essential oils and hydrosols from muscadine flowers and berry skins of both Carlos and Noble cultivars (Figure 2).

The analyses of the data showed that the first five principal components had eigenvalues greater than 1 (Figure 2a). These five components explain 97.9% of the variation in the data. Figure 2b shows the score plot of the first two principal components. The first principal component (PC1) had an eigenvalue of 18.385 and explained 44.8% of the variation in the data, whereas the second principal component (PC2) had an eigenvalue of 12.608 and explained 30.8% of the variation in the data. PC1 had large positive associations with β-linalool and *trans-*geraniol, whereas PC2 had large positive associations with 4-hydroxy-3-methylacetophenone, 3,4,5-trimethoxytoluene, 1,3,5-trimethyoxybenzene, elemicin, nerolidol, ledol, globulol, *epi*-α-cadinol, *epi*-α-muurolol, torreyol, α-cadinol, and juniper camphor. As is shown in Figure 2b, by PC1 it was possible to distinguish the essential oils from flowers of both cultivars from their hydrosols and the essential oils and hydrosols from berry skins. However, the separation among essential oils and hydrosols obtained from berry skins of Noble and Carlos muscadine cultivars was unclear.

### 2.2. Evaluation of Antioxidant Potential

The antioxidant potential of essential oils and hydrosols from flowers and berry skins of *M. rotundifolia* (Michx.) Small. Noble and Carlos cultivars was evaluated using five in vitro methods based on different reaction mechanisms: two free radical scavenging methods, utilizing both single electron transfer and hydrogen atom transfer reaction mechanisms (DPPH and TEAC), two ion reducing power methods, utilizing only the single electron transfer reaction mechanism (FRAP and CUPRAC), and the nitric oxide radical scavenging activity (NO), utilizing the sequential proton loss electron transfer reaction mechanism [27,28]. Among these methods, the DPPH and TEAC methods were characterized by the lowest specificity to the nature of the antioxidants, but with good sensitivity and high repeatability. The CUPRAC and FRAP methods were more selective, highly sensitive, and more accurate, since they are based on a single electron reaction mechanism, whereas the NO scavenging activity assay had the lowest detection limit. The data are presented in Table 2. In general, the essential oils showed much stronger antioxidant potential when compared with the hydrosols. The essential oil from Carlos flowers showed the strongest antioxidant activities in all assays, followed by the essential oil from flowers of Noble (Table 2). Both oils were significantly stronger antioxidants (*p* ≤ 0.01) than the positive control (gallic acid) (Table 2). All oils showed significantly weaker ability to scavenge the DPPH radical when compared to gallic acid (DPPH value of 15,004.86 ± 43.05). This could be explained by the fact that the assay is based on a non-competitive reaction, and DPPH itself could serve both as a radical probe and oxidant, and thus, many antioxidants may react slowly and may even be inert to DPPH radical [28]. The observed high values of the CUPRAC assay were probably due to the running process of redox cycling because of the lowest redox potential of the CUPRAC system, which could be considered an indication for the presence of compounds with pro-oxidant action in these oils [28]. The four essential oils showed a significantly higher capacity (*p* ≤ 0.01) to scavenge the nitric oxide radical than the positive control (gallic acid). Moreover, the essential oils from berry skins showed the highest nitric oxide scavenging activities, with EC_50_ = 10.0 ± 0. 2 mg for Carlos and EC_50_ = 10.0 ± 0. 4 mg for Noble berry skin oils (Table 2). Controlling the level of nitric oxide is a very important physiological process, since it has signaling, regulatory, and protective functions. However, when the local concentration of nitric oxide is increased under certain conditions, it can initiate the formation of nitrogen oxide species such as nitroxyl and peroxynitrite radicals, which can damage biological molecules [29]. The control of nitric oxide levels is very important in dermatology; this compound has been associated with erythema, melanogenesis, inflammatory skin disease, and skin cancer [29,30]. Recently, it has been reported that nitric oxide is an important part of invertebrate defense mechanisms against parasites, viruses, and bacteria [31].

The observed abilities of muscadine essential oils to scavenge nitric oxide and ABTS radicals, and to reduce ferric and cupric ions, outlines the potential of these oils as prospective antioxidant additives in cosmetics and skin care products.

### 2.3. Evaluation of Antimicrobial Activity

For the evaluation of antimicrobial activities, the two essential oils from flowers as well as four hydrosols from the flowers and berry skins of Noble and Carlos cultivars were tested. The low yields the essential oils from Carlos and Noble berry skins (0.012 ± 0.0001% (*w*/*w*) and 0.010 ± 0.0007% (*w*/*w*), respectively) were not enough to perform reliable biological experiments.

The antimicrobial activities were tested against two Gram-positive bacteria (*Bacillus cereus* ATCC 11778 and *Staphylococcus aureus* ATCC 6538), two Gram-negative bacteria (*Escherichia coli* ATCC 8739 and *Pseudomonas aeruginosa* ATCC 9027), and one dimorphic yeast (*Candida albicans* ATCC 10231). The results are presented in Table 3. None of the hydrosols showed any antimicrobial effect. Both essential oils from muscadine flowers showed low antibacterial activity against Gram-positive bacteria (MIC = 1.0 mg/mL) and no activity against Gram-negative bacteria (Table 3). Interestingly, both oils showed moderate antifungal activities against *Candida albicans* ATCC 10231 (Table 3). This could be explained by the high concentrations of the sesquiterpene germacrene D, which has well known antifungal properties [32,33]. Moreover, the antifungal activity of oil from Carlos flowers (MIC = 0.125 mg/mL) was twice as strong as that of the oil from Noble flowers (MIC = 0. 25 mg/mL), which corresponded with the almost twice higher concentrations of germacrene D, α-selinene, and α-cadinol detected in this cultivar (Table 1). This is the first report on evaluation of antimicrobial activities of essential oils and hydrosols obtained by hydro-distillation of grape flowers. However, for complex evaluation of the antimicrobial potential of grape volatile compounds, more research and experiments with different grape varieties and test microorganisms must be performed.

## 3. Materials and Methods

### 3.1. Plant Material

Flowers and berry skins of red (*Muscadinia rotundifolia* (Michx.) Small., cultivar Noble) and yellow (*Muscadinia rotundifolia* (Michx.) Small., cultivar Carlos) muscadine grapes were collected from the experimental vineyards of the Center for Viticulture and Small Fruit Research in the College of Agriculture and Food Sciences at Florida Agriculture and Mechanical University in Tallahassee, Florida (30°28′32.9″N 84°10′25.9″W). The flowers were collected in May 2015 from 20 vines (10 vines of Noble and 10 vines of Carlos cultivars), randomly chosen in different rows. The plants were marked and used later to collect ripe berries. The berries were collected in August 2015 for the Noble cultivar and in September 2015 for the Carlos cultivar. The maturity of the berries was evaluated on the basis of their pH, sugar content, and titratable acidity. Berries were picked randomly from different bunches throughout the vines. Samples were processed immediately after collection. The pulps were pulled out and the berry skins were collected for distillation of essential oils.

### 3.2. Essential Oils and Hydrosols

Five hundred grams of fresh flowers and one kilogram of fresh berry skins from both cultivars were used for obtaining essential oils and their corresponding hydrosols. The essential oils were obtained by water distillation in a Clevenger-type laboratory glass apparatus (VWR International, Atlanta, GA, USA) according to British Pharmacopoeia, modified as described elsewhere [34]. The moisture of the grape flowers and berry skins was determined with a moisture analyzer (Mettler Toledo MJ33 Moisture Determination Balance, Mettler-Toledo, LLC, Columbus, OH, USA) using the standard protocol of the apparatus up to obtaining a constant weight. Distillation was performed for 4 h. The oils and hydrosols were stored at −20 °C prior the analyses.

### 3.3. GC-MS Analyses

Before gas chromatography analyses, the volatiles from hydrosols (50 mL) were extracted with *n*-hexane (Sigma-Aldrich, Merck KGaA, Darmstadt, Germany) (3 × 2 mL). After separation of phases, the combined hexane fractions were dried over anhydrous sodium sulfate, filtered, and used for GC-MS analyses. The GC-MS analyses were performed on an Agilent Technology Hewlett Packard 7890 A+/MSD 5975 (Hewlett Packard, Palo Alto, CA, USA) gas chromatograph, coupled with an Agilent Technology 5975C inert XL EI/CI MSD (Hewlett Packard, Palo Alto, CA, USA) mass spectrometer. A HP-5MS column (30 m × 250 μm × 0.25 μm) was used. The column temperature was maintained at 60 °C for 2 min, increased to 260 °C at 5 °C per minute, and held at 260 °C for 8 min. The injection volume was 1 μL, and a split ratio of 10:1 was used. The injector temperature was set up to 250 °C. The flow rate of carrier gas (helium) was 1 mL/min. The MS source was setup to 230 °C, and MS quad was 150 °C. EI/MS were recorded at 70 eV. The retention indices (RI) of the compounds were recorded with a standard *n*-hydrocarbon calibration mixture (C9–C36) (Restek, Teknokroma, Spain) using AMDIS 3.6 software. The compounds in essential oils and hydrosols were identified by their calculated RI (Kovats) and mass spectra, compared with those of reference compounds included in the Wiley/NIST database or specialized literature data [35]. The results were expressed as relative percentages of the total ion current (TIC).

### 3.4. Antioxidant Activity Assays

DPPH assay was performed as described elsewhere [36] with some modifications. Briefly, 2.85 mL of 0.1 mM solution of DPPH (1,1-diphenyl-2-picrylhydrazyl radical, Sigma-Aldrich) in methanol (Sigma-Aldrich) was mixed with 0.15 mL of sample (essential oils were dissolved in methanol, whereas hydrosols were tested directly). The reaction was performed for 15 min, at 37 °C in darkness, and the decrease in the absorbance (517 nm) was measured against methanol (Shimadzu UV-Vis 1240, Shimadzu Corp., Kyoto, Japan). Trolox (6-hydroxy-2,5,7,8-tetramethylchroman-2-carboxylic acid, Sigma-Aldrich) solutions at concentrations of 0.1; 0.2; 0.3; 0.4, and 0.5 mM were used to build the standard calibration curve. The antioxidant activity was expressed as mM Trolox equivalents (TE) per gram of essential oil.

TEAC assay was performed as described elsewhere [36] with some modifications. Briefly, ABTS radical was generated by mixing equal aliquots of 7 mM solution of ABTS (2,2′azinobis (3)-ethylbenzthiazoline-6-sulfonic acid, Sigma-Aldrich) in H_2_O and 2.45 mM solution of potassium persulfate (Sigma-Aldrich) in H_2_O for 16 h. Next, 2.85 mL of freshly diluted ABTS^+^ solution was mixed with 0.15 mL of sample (essential oils were dissolved in methanol, whereas hydrosols were tested directly). The reaction was performed for 15 min, at 37 °C in darkness, and the decrease in the absorbance (734 nm) was measured against methanol (Shimadzu UV-Vis 1240 spectrophotometer). Trolox solutions at concentrations of 0.1, 0.2, 0.3, 0.4, and 0.5 mM were used to build the standard calibration curve. The antioxidant activity was expressed as mM TE per gram of essential oil.

FRAP assay was performed as described elsewhere [36] with some modifications. Briefly, 3 mL of freshly prepared FRAP reagent (10 parts of 300 mM sodium acetate buffer with pH 3.6; 1 part of 10 mM TPTZ (2,4,6tripyridyl-s-triazine, Sigma-Aldrich) solution in 40 mM HCl and 1 part of 20 mM iron(III) chloride hexahydrate (Sigma-Aldrich) solution in H_2_O) was mixed with 0.1 mL of sample (essential oils were dissolved in methanol, whereas hydrosols were tested directly). The blank sample was prepared by the same way, but without the addition of antioxidant. The reaction was performed for 4 min at 37 °C, and the absorbance (593 nm) of the investigated samples was read against the absorbance of the blank (Shimadzu UV-Vis 1240 spectrophotometer). Trolox solutions at concentrations of 0.1, 0.2, 0.3, 0.4, and 0.5 mM were used to build the standard calibration curve. The antioxidant activity was expressed as mM TE per gram of essential oil.

CUPRAC assay was performed as described elsewhere [36] with some modifications. Briefly, 1.0 mL 10 mM copper dichloride hydrate (Sigma-Aldrich) was mixed with 1.0 mL 7.5 mM neocuproine (Sigma-Aldrich), 1 mL 1 M ammonium acetate buffer (pH 7.0), 0.1 mL of investigated sample (essential oils were dissolved in methanol, whereas hydrosols were tested directly), and 1.0 mL H_2_O. The blank sample was prepared by the same way, but without the addition of antioxidant. The reaction was performed for 20 min at 37 °C, and the changes in absorbance (450) nm were measured against the absorbance of the blank (Shimadzu UV-Vis 1240 spectrophotometer). Trolox solutions at concentrations of 0.1, 0.2, 0.3, 0.4, and 0.5 mM were used to build the standard calibration curve. The antioxidant activity was expressed as mM TE per gram of essential oil.

Nitric oxide (NO) radical scavenging activity was measured as described elsewhere [37] with some modifications. Briefly, 1.0 mL of different concentrations of sample (essential oils were dissolved in methanol, whereas hydrosols were tested directly) was mixed with 1.0 mL 5 mM sodium nitroprusside (Sigma-Aldrich) solution in PBS (phosphate buffer saline). A blank sample containing all reagents except for the test sample was prepared as well. The mixtures were incubated for 150 min at 25 °C, and then an equal aliquot of Griess reagent (Sigma-Aldrich) was added. The absorbance (546 nm) of the formed chromophore was measured for both blank and tested samples (Shimadzu UV-Vis 1240 spectrophotometer), and the percent of NO inhibition was calculated for each sample concentration. The data were used for expression of antioxidant activity as half effective concentrations (EC_50_).

Gallic acid was used as a positive control for each antioxidant method used.

### 3.5. Antimicrobial Activity Evaluation

The antimicrobial effects of the essential oils and hydrosols were tested against the Gram-positive bacteria *Bacillus cereus* ATCC 11778 and *Staphylococcus aureus* ATCC 6538, as well as the Gram-negative bacteria *Escherichia coli* ATCC 8739 and *Pseudomonas aeruginosa* ATCC 9027, and the dimorphic yeast *Candida albicans* ATCC 10231. All strains were deposited in the Microbial Culture Collection of the Department of Biochemistry and Microbiology (University of Plovdiv, Bulgaria). The bacterial strains were stored on nutritional agar (NA-HiMedia Ltd., HiMedia Laboratories GmbH, Einhausen, Germany.) and the yeast strains on Sabouraud dextrose agar with chloramphenicol (SDA, HiMedia Ltd.). Stock solutions of the samples for antimicrobial testing were prepared by dissolving the respective compound in 2% DMSO (Sigma-Aldrich Co.). Antibacterial activity of the samples was assessed according to Clinical Laboratory Standard Institute (CLSI) M2-A9 reference method for antimicrobial disk susceptibility tests [38] and CLSI M7-A7 reference method for dilution antimicrobial susceptibility tests for bacteria that grow aerobically [39]. Anticandidial activity of essential oils was performed according to CLSI M44-A2 reference method for antifungal disk diffusion susceptibility testing of yeasts [40] and CSLI M27-A3 reference method for broth dilution antifungal susceptibility testing of yeasts [41]. Controls consisting of inoculated medium without tested sample and without DMSO, as well as with DMSO, were also prepared. The DMSO concentration in the broth dilution assay was low to keep the effect on microbial growth to a minimum. Antimicrobial activity determined by broth microdilution tests was expressed as minimal inhibitory concentration (MIC) in µg/mL. MIC was defined as the lowest concentration of the tested compound at which total inhibition of microbial growth was detected. Antimicrobial activity of essential oils determined by disc diffusion tests was expressed as inhibitory zone diameter in mm (IZ, mm). IZ diameters were measured to the nearest millimeter by antibiotic zone scale (HiMedia Laboratories Ltd.). Antimicrobial activities of ciprofloxacin (CPH 5µg/disc, HiMedia Laboratories Ltd.) and fluconazole (FLC 25µg/disc, HiMedia Laboratories Ltd.) were also determined and used as positive controls.

### 3.6. Data Analysis

All the analyses were performed in triplicate with three independent technical measurements. The results are expressed as mean values with standard deviations (±SD) (*n* = 3). The means were statistically compared using one-way ANOVA with Tukey pairwise comparison test. The Dunnett multiple comparisons with a control was used to compare the means with the positive control. The differences between the means were considered significant for values of 𝑝 ≤ 0.01. The statistical tests were performed using MiniTab 17 Statistical Software (Minitab INC, State College, PA, USA).

## 4. Conclusions

This study reported for the first time the chemical composition, antioxidant potential, and antimicrobial properties of essential oils and hydrosols from flowers and berry skins of two muscadine cultivars: the red *M. rotundifolia* cultivar Noble, and the yellow *M. rotundifolia* cultivar Carlos. The major volatile compounds found in the essential oils from muscadine flowers were valencene, germacrene D, α-selinene, and α-cadinol, whereas the major identified compounds in essential oils from muscadine berry skins and hydrosols were α-terpineol and β-linalool. All of the obtained essential oils showed a much higher antioxidant potential than the hydrosols. The oils showed increased potential to reduce ferric and cupric ions and to scavenge nitric oxide and ABTS radicals. However, for a complete evaluation of the antioxidant potential of muscadine volatiles, additional experiments involving in vivo assays for evaluation of their antioxidant activities have to be performed in future research. The essential oils, obtained from the flowers of both muscadine cultivars, showed moderate antifungal activities against *Candida albicans*. To the best of our knowledge, this is the first report to obtain and characterize essential oils and hydrosols from muscadine grapes. This study demonstrated the existence of important variations in the aromatic compounds accumulated in the flowers and mature berry skins of muscadine grapes. The presented results will be the base for future research focused on better understanding of the physiological role of aromatic compounds in muscadine vines, and could be considered the first step in study of volatile compound biosynthesis and accumulation in muscadine grapes.

## Figures and Tables

**Table 1:** Chemical composition of essential oils and hydrosols from two *M. rotundifolia* (Michx.) Small. cultivars.

Compound	Calculated Retention Index (Kovats)	% of TIC							
		EO *, Car-F	EO, Nob-F	EO, Car-B	EO, Nob-B	HY **, Car-F	HY, Nob-F	HY, Car-B	HY, Nob-B
Eucalyptol	1031	-	-	1.28	0.15	-	-	-	-
β-Linalool	1097	0.29	0.11	8.69	5.95	12.44	10.73	7.62	6.50
Myrcenol	1117	-	-	5.10	0.08	-	-	-	-
*allo*-Ocimene	1130	-	-	14.05	0.23	-	-	-	-
β-Terpineol	1145	-	-	1.17	-	-	-	5.33	6.29
*p*-Cymen-8-ol	1184	-	-	-	-	-	-	13.38	7.08
α-Terpineol	1190	0.18	0.07	45.42	59.43	10.39	12.22	65.41	72.83
*cis*-Geraniol	1227	-	-	0.86	0.10	1.21	0.15	1.29	1.20
*trans*-Geraniol	1256	-	-	1.38	0.96	2.97	1.58	1.17	0.88
4-Hydroxy-3-methyl acetophenone	1322	-	-	-	-	6.56	18.94	-	-
α-Cubebene	1349	0.13	0.15	-	-	-	-	-	-
Eugenol	1355	-	-	1.69	0.08	-	-	1.03	-
Ylangene	1371	0.27	0.69	-	-	-	-	-	-
α-Copaene	1376	0.41	0.35	-	-	-	-	-	-
*trans*-β-Damascenone	1381	-	-	0.87	0.09	-	-	-	-
3,4,5-Trimethoxy toluene	1398	-	-	-	-	4.65	7.42	-	-
1,3,5-Trimethyoxy benzene	1416	-	-	-	-	3.09	5.04	-	-
β-Caryophyllene	1419	3.48	3.72	-	-	-	-	-	-
(+)-Aromadendrene	1439	3.01	3.49	-	-	-	-	-	-
β-Farnesene	1444	2.79	3.72	-	-	-	-	-	-
α-Humulene	1455	1.64	1.45	-	-	-	-	-	-
*allo*-Aromadendrene	1462	2.74	3.11	-	-	-	-	-	-
Germacrene D	1479	6.94	4.48	-	-	-	-	-	-
β-Selinene	1486	3.43	3.75	-	-	-	-	-	-
Valencene	1490	34.32	39.71	-	-	-	-	-	-
α-Selinene	1495	4.29	3.28	-	-	-	-	-	-
α-Farnesene	1505	2.26	1.29	-	-	-	-	-	-
α-Selinene, 7-epi	1517	3.28	2.03	-	-	0.35	2.69	-	-
α-Cadinene	1539	2.16	2.88	-	-	-	-	-	-
Elemicin	1555	-	-	-	-	1.23	0.09	-	-
Nerolidol	1564	-	-	-	-	0.35	0.12	-	-
Ledol	1565	-	-	-	-	0.46	0.25	-	-
Globulol	1585	1.53	0.82	-	-	3.92	0.60	-	-
Veridiflorol	1589	-	-	0.29	0.65	0.22	0.16	-	-
Humulene epoxide II	1606	2.21	1.42	-	-	0.29	2.97	-	-
Asarone	1623	-	-	-	-	2.32	0.10	-	-
*epi*-α-Cadinol	1641	1.03	0.68	-	-	2.85	4.36	-	-
*epi*-α-Muurolol	1643	1.45	1.08	-	-	8.77	6.18	-	-
Torreyol	1646	1.64	0.54	-	-	3.00	2.74	-	-
α-Cadinol	1654	4.30	2.86	-	-	22.56	14.26	-	-
Juniper camphor	1690	-	-	-	-	5.83	3.19	-	-

Car-F—Carlos flowers; Car-B—Carlos berry skins; Nob-F—Noble flowers; Nob-B—Noble berry skins; * EO—essential oil; ** HY—hydrosol.

**Table 2:** Antioxidant activities of essential oils and hydrosols from two *M. rotundifolia* (Michx.) Small. cultivars.

Sample	DPPH, µM Trolox Eq./g Oil	TEAC, µM Trolox Eq./g Oil	FRAP, µM Trolox Eq./g Oil	CUPRAC, µM Trolox Eq./g Oil	NO, EC_50_ **, mg Oil; µL Hydrosol
EO, Car-F	3173.7 ± 326.4 *^, a^	112,986.5 ± 742.3 *^, a^	56,286.3 ± 466.5 *^, a^	1,141,694.4 ± 2455.9 *^, a^	20.0 ± 0.1 *^, d^
EO, Nob-F	2549.3 ± 308.8 *^, a,b^	79,276.3 ± 431.7 *^, b^	43,884.9 ± 336.9 *^, b^	878,509.6 ± 1901.9 *^, b^	20.0 ± 0.1 *^, d^
EO, Car-B	2964.7 ± 116.5 *^, a^	4239.0 ± 162.9 *^, c^	12,400.8 ± 160.0 *^, c^	649,043.4 ± 1753.3 *^, c^	10.0 ± 0.2 *^, d^
EO, Nob-B	1712.0 ± 256.8 *^, b^	1382.3 ± 108.9 *^, d^	7964.2 ± 352.5 *^, d^	575,580.6 ± 2160.2 *^, d^	10.0 ± 0.0 *^, d^
HY, Car-F	33.5 ± 0.7 *^, c^	71.8 ± 2.3 *^, e^	22.1 ± 0.6 *^, e^	1.2 ± 0.0 *^, e^	1720.0 ± 0.1 *^, c^
HY, Nob-F	39.6 ± 2.2 *^, c^	81.0 ± 0.8 *^, e^	28.6 ± 1.0 *^, e^	14.3 ± 2.6 *^, e^	2030.0 ± 0.2 *^, b^
HY, Car-B	21.0 ± 1.5 *^, c^	20.2 ± 1.3 *^, e^	19.9 ± 1.0 *^, e^	1.2 ± 0.0 *^, e^	1700.0 ± 0.2 *^, c^
HY, Nob-B	11.6 ± 0.7 *^, c^	10.5 ± 0.8 *^, e^	17.9 ± 1.0 *^, e^	2.1 ± 1.5 *^, e^	2220.0 ± 0.2 *^, a^
Positive Control (Gallic Acid)	15,004.9 ± 43.1	23,297.7 ± 25.3	14,850.4 ± 77.4	13,418.2 ± 160.4	210.0 ± 0.4

* Means are significantly different from the control level mean (Dunnett multiple comparisons with a control at 99.9% confidence); means that do not share a letter in superscript in the column are significantly different (Tukey pairwise comparisons, 𝑝 value ≤ 0.01). ** EC_50_—half maximal effective concentration.

**Table 3:** Antimicrobial activities of essential oils and hydrosols from two *M. rotundifolia* (Michx.) Small. cultivars.

Test Microorganism	Essential Oil, Flowers, Carlos		Essential Oil, Flowers, Noble		Positive Control	
	IZ ± SD *, mm	MIC **, % (*w*/*v*)	IZ ± SD, mm	MIC, % (*w*/*v*)	IZ ± SD, mm	MBC/MFC *** µg/mL
*Staphylococcus aureus* ATCC 6538	10.06 ± 0.12	1.00	9.23 ± 0.25	1.00	31.30 ± 0.29	0.125
*Bacillus cereus* ATCC 11778	8.23 ± 0.23	1.00	8.06 ± 0.12	1.00	28.30 ± 0.30	0.125
*Escherichia coli* ATCC 8739	8.06 ± 0.12	2.00	8.06 ± 0.12	2.00	21.00 ± 0.28	0.25
*Pseudomonas aeruginosa* ATCC 9027	-	>2.00	-	>2.00	9.60 ± 0.17	1.00
*Candida albicans* ATTC 10231	14.20 ± 0.32	0.125	12.30 ± 0.26	0.25	16.60 ± 0.29	0.25

* IZ—inhibitory zone diameter (mean ± standard deviation, *n* = 3); ** MIC—minimal inhibitory concentration; *** MBC—minimal bactericidal concentration; MFC—minimal fungicidal concentration.
